# Editorial: Drug development for pancreatic cancer: novel targets, drug candidates and mechanisms

**DOI:** 10.3389/fonc.2025.1705678

**Published:** 2025-10-10

**Authors:** Xiao Sun, HongDe Xu

**Affiliations:** ^1^ Tianjian Laboratory of Advanced Biomedical Sciences, Academy of Medical Sciences, Zhengzhou University, Zhengzhou, China; ^2^ School of Pharmaceutical Sciences, Zhengzhou University, Zhengzhou, China

**Keywords:** pancreatic cancer, pancreatic ductal adenocarcinoma, CAR-T cells, natural products, SUMOylation

Pancreatic ductal adenocarcinoma (PDAC), the most prevalent subtype of pancreatic cancer, constitutes approximately 90% of all pancreatic cancer cases. It is recognized as one of the most aggressive malignancies globally, with a five-year survival rate persistently around 12% (1). The absence of early specific symptoms and effective screening methods results in over 80% of patients being diagnosed at an advanced stage, frequently with local invasion or distant metastasis, thereby significantly limiting treatment options and contributing to a poor prognosis (2). This challenging scenario highlights the urgent necessity to enhance our understanding of the biological mechanisms underlying PDAC and to develop innovative therapeutic strategies.

While engineered T cell therapy has demonstrated significant success in treating hematologic malignancies, its application in PDAC remains fraught with substantial challenges (3). These include an immunosuppressive microenvironment, tumor antigen heterogeneity, and inadequate T cell infiltration coupled with functional exhaustion. Moreover, PDAC treatment is hindered by several factors: a highly heterogeneous genomic landscape, a complex tumor microenvironment (TME), a persistently immunosuppressive state, and metabolic reprogramming mechanisms, all of which contribute to therapy resistance. Addressing these challenges necessitates interdisciplinary collaboration and the development of integrated, multi-dimensional interventional strategies—this is the primary research focus that this Research Topic seeks to advance.

This collection of studies presents four pioneering investigations that illuminate the biological characteristics and therapeutic strategies of PDAC from diverse perspectives. These studies not only advance the understanding of disease mechanisms but also investigate novel interventions with translational potential, including immune cell engineering, natural product screening, and epigenetic regulation. Notably, the research conducted by Zhu et al. transcends traditional αβ T cell approaches by concentrating on the application of γδ T cells and natural killer T (NKT) cells in PDAC immunotherapy. Utilizing a mesothelin/CD3 bispecific antibody (BsAb), the authors demonstrate that these cells exhibit significantly enhanced tumor-killing activity both *in vitro* and *in vivo*, while substantially reducing the release of inflammatory cytokines such as IL-6 and IL-1β. This reduction in cytokine release mitigates the toxicities commonly associated with conventional αβ T cell-based therapies. Consequently, this study broadens the spectrum of cellular immunotherapy options and provides new pathways for enhancing treatment safety.

Chen et al. tackle the issue of CAR-T cell exhaustion in PDAC through a novel strategy involving the co-expression of the IL-15/IL-15Rα complex (IL15C). This approach activates the JAK3/STAT5 signaling pathway, thereby significantly enhancing the proliferative capacity and anti-apoptotic characteristics of NKG2D-CAR T cells. The engineered cells exhibit a marked reduction in the expression of exhaustion markers, such as PD-1 and TIM-3, and preferentially differentiate into central memory T cells, leading to more sustained tumor control *in vivo*. This innovative strategy offers a valuable framework for optimizing CAR-T cell therapy in the context of solid tumors.

In parallel, although Shao et al. conducted their investigation within a lung cancer model, their comprehensive research methodology offers substantial insights for PDAC drug development. By integrating network pharmacology, molecular docking, and functional validation, the researchers elucidate the anti-tumor mechanisms of cyclomorusin, an active compound derived from the traditional Chinese medicine Cortex Mori. This compound exerts its effects by inhibiting the PI3K/AKT/mTOR signaling pathway. The study highlights the contemporary scientific relevance of compounds derived from traditional medicine and provides a theoretical basis for the development of natural products targeting pancreatic ductal adenocarcinoma.

Wang et al. explore the role of protein SUMOylation in PDAC and develop a prognostic scoring system (Sscore) based on SUMO substrate-encoding genes, including CDK1, AHNAK2, and SAFB. This model demonstrates efficacy in predicting patient survival and shows associations with genomic instability, immune infiltration, and drug sensitivity. Notably, through functional experiments, the authors identify SAFB2 as a novel tumor suppressor that impedes PDAC progression by inhibiting the Wnt/β-catenin pathway. This study offers a dual theoretical foundation for SUMOylation-targeted therapy and personalized prognostic evaluation.

The studies featured in this topic collectively encompass multiple dimensions, ranging from fundamental mechanisms to translational applications, underscoring the significance of integrated multi-strategy interventions in PDAC treatment. Each article not only presents novel experimental evidence but also expands therapeutic perspectives. Whether through the diversification of immune cell types, the application of cytokine engineering, multi-target regulation via natural compounds, or mechanistic insights at the epigenetic level, these contributions reflect the field’s diversity and innovative vitality.

The studies featured in this Topic collectively cover multiple dimensions—from fundamental mechanisms to translational applications—highlighting the importance of combined multi-strategy interventions in PDAC treatment. Each article not only presents novel experimental evidence but also broadens therapeutic perspectives. Whether through the expansion of immune cell types, the application of cytokine engineering, multi-target regulation via natural compounds, or mechanistic insights at the epigenetic level, these contributions reflect the diversity and innovative vitality of the field. However, determining the most effective treatment strategy for patients, systematically evaluating the advantages and disadvantages of various therapeutic approaches, and ultimately developing a personalized precision treatment framework remain critical challenges that demand urgent attention in future research.
